# 

**Published:** 1887-06

**Authors:** 


### TABLE OF CONTENTS.

### Original and Selected Articles.

Notes of Professor Gaston's Lecture on Malignant Tumors, by J. E Kintz, M. D., Somerset, Ohio (concluded) ..... 221
Articles of Food that are Laxative; To Reduce Weight ..... 226
Modification of MacEwing's Operation for the Radical Cure of Hernia, by Chas. N. Jones, M. D., Brooklyn. N. Y ..... 227
To Increase Weight ..... 229
The Work of Micro-Organisms, by Dr. Hiram Christopher, of Missouri ..... 230
The Treatment of Hysterical Attacks; Pyridine in Asthma ..... 238

### Abstracts and Gleanings.

Treatment of Hemorrhagic Malarial Fever ..... 239
The Subiodide of Bismuth in the Treatment of Ulcerations; Triple Contagion of Tuberculosis—from Man to Man, from Man to Fowls, and from Fowls to Man ..... 241
Measles ..... 242
Dangers of Santonin; A Speedy Cure of Whooping Cough ..... 243
Cold Bathing; Point of Diagnosis in Rotheln; Management of Simple Constipation ..... 244
The Rational Treatment of Patients After Cataract Operations ..... 246
Clergyman's Sore Throat; Treatment of Chronic Ulcers ..... 247
The Cure of Large Ulcers of the Leg by the Carbolized Spray ..... 248
Inoculation as a Preventive of Yellow Fever; The Etiology of Dysentery ..... 249
The Use of Spleen-Pulp in Anæmia; Drumin 250

### Scientific Items.

On Sugar in the Blood; Does Consciousness Continue after Decapitation ..... 251
Asbestos Paper; A Wonderful Advance in the Application of Electricity ..... 252

### Practical Notes and Formulæ.

For Cholera Morbus. etc; Cannabis Indica in Acute and Chronic Dysentery; To Stop Toothache; Prescription for Headache ..... 253
Rheumatism; Earache; A Laxative and Tonic Wine; Cocaine Cotton ..... 254
Acute Dysentery; Chills; Cocaine in the Treatment of Dysentery ..... 255

### Editorials and Miscellaneous.

To our subscribers; Editorial Brevities ..... 256
A Unique Message; Inebriety; Nullification in an Eclectic; A Dental School in Atlanta ..... 257
East Tennessee, Virginia and Georgia Railroad Co.; Stone Mountain Medical Association ..... 258
The International Medical Congress ..... 260
Duality of the Brain—Mind Reading ..... 261
The Microbe of Yellow Fever ..... 262
Receipted; Special Notes ..... 264

